# Utilization of telerehabilitation in TKR patients: A systematic review

**DOI:** 10.1371/journal.pone.0324074

**Published:** 2025-07-23

**Authors:** Farnaz Salehian, Jahanpour Alipour, Somayyeh Zakerabasali

**Affiliations:** 1 Student Research Committee, Department of Health Information Management, School of Health Management and Information Sciences, Shiraz University of Medical Sciences, Shiraz, Iran; 2 Department of Health Information Management, Health Human Resources Research Center, School of Health Management and Information Sciences, Shiraz University of Medical Sciences, Shiraz, Iran; 3 Clinical Education Research Center, Health Human Resources Research Center, School of Health Management and Information Sciences, Shiraz University of Medical Sciences, Shiraz, Iran; Neighborhood Physical Therapy, UNITED STATES OF AMERICA

## Abstract

**Introduction:**

Advanced telerehabilitation technology helps physiotherapists monitor the patient’s treatment process after Total Knee Replacement. This study aimed to review the research on telerehabilitation after Total Knee Replacement, synthesize the findings related to their applications and features, and address evaluations made on them.

**Methods:**

A systematic search was conducted in PubMed, Scopus, and Web of Science from 2019 through 2023. The authors selected the articles based on keywords and criteria and reviewed them in terms of title, abstract, and full text. Full-text articles and English language, focusing on telerehabilitation for TKR patients, preferably including mobile health applications and consistent with the research question, were considered for more review. Then, the MMAT (mixed methods appraisal) tool was used to assess each article. Finally, the selected articles were evaluated. The systematic review was registered through PROSPERO with registration ID: PROSPERO CRD42024533040.

**Result:**

After reviewing databases, 183 articles were retrieved, then 84 duplicate articles were removed. Of the remaining 99 articles, 40 were deleted after reviewing the title and abstract because they were grey literature or irrelevant to TKR surgery, and 41 were deleted after reviewing the full text. Finally, 18 articles were included in this study and analyzed. The United States was the most common country that developed a telerehabilitation system. The most common use of this technology has been in education, treatment, and subsequent monitoring. Sensor and wearable activity trackers are the most common equipment used in studies. The most common study designs were randomized controlled trials (RCT) and observational studies, each of which accounted for 9 (50%) and 4 (22.22%), respectively.

**Conclusion:**

Telerehabilitation can be as effective as traditional rehabilitation in improving the condition of patients after TKA. However, it is suggested that improvements should always be made to achieve better results of telerehabilitation. Most of the TKA apps in the reviewed studies showed significant effectiveness. Information and communication technology are used to provide high-quality, low-cost, continuous treatments. Soon, telerehabilitation will play a more prominent role and will be more popular.

## Introduction

Total knee replacement (TKR) is one of the treatment options considered one of the best surgical treatment options available and leads to relieving the patient’s pain and increasing functional results. This method also significantly increases patient satisfaction by more than 80%. With increasing expectations for a physically active lifestyle and increasing demand for surgery at a younger age, the importance of this surgery is increasing daily [1]. The average age of TKR patients in the world is decreasing. In other words, the demand for surgery in younger people (under 65) is increasing and is expected to double in the next decade [2].

TKR is a one-day operation in which patients do not need to stay overnight in the hospital, but full recovery after surgery takes an average of 6–12 months. Most TKR patients report difficulty moving and limited physical activity after surgery, which is caused by pain, joint stiffness, and insufficient muscle strength. Early postoperative mobility has been shown to improve functional mobility and prevent deep vein thrombosis; therefore, it is important to start the rehabilitation program at this time [3].

Postoperative physiotherapy is standard, but adherence to physiotherapy programs can be low. Adherence can be low due to pain during exercise, not being very active before surgery, or due to social and psychological issues. Advanced telerehabilitation technology through mobile health applications helps physiotherapists monitor the patient’s treatment process. In this way, the therapist can ensure that the exercises are performed correctly, increasing motivation and thus improving patient adherence. Using rehabilitation programs through mobile applications may be a time-efficient and cost-effective alternative to regular clinical sessions [4].

Recently, mobile applications have played an important role in monitoring and motivating patients to participate in rehabilitation programs. Telerehabilitation through Mobile Health (mHealth) programs aim to increase patient participation and build self-management capabilities for postoperative health care [5]. Despite the importance of physiotherapy and exercise therapy after knee replacement surgery and the role of mobile health in facilitating the treatment process, few systematic reviews have been conducted in this field [6–8]. Therefore, the present study is mainly aimed at investigating the role of telerehabilitation in helping patients after knee joint replacement and a general review of their applications and features.

## Method

### Search design

This systematic review follows the preferred reporting items for systematic reviews and meta‐analyses (PRISMA) guidelines for identifying potentially related articles to total knee arthroplasty mobile health. The study protocol was registered with the International Prospective Register of Systematic Reviews (PROSPERO) in accordance with PRISMA-P guidelines (PROSPERO CRD42024533040) before starting the study process. We conducted this systematic review by searching PubMed, Web of Science, and Scopus’s online databases for relevant literature using keywords and operational phrases. The keywords are written in Table 1, and the search strategy is written in the S1 Appendix. The search was conducted between January 1, 2019, and November 30, 2023. We removed duplicates and applied a two-stage filtering process. Initially, we screened titles and abstracts to exclude irrelevant articles and then assessed the full texts of remaining studies against predetermined inclusion and exclusion criteria. Our search terms were derived from PubMed’s Mesh Term section and relevant studies. The PICO framework (population, Intervention, comparator, and outcome) was used to structure the research question: “What are the applications and features of Mobile health applications for patients after total knee replacement?” The population includes people who have total knee replacement. The Intervention provides Mobile health applications that facilitate telerehabilitation services for this group of patients. The comparator is not considered in this study. The outcome is that the mobile health application has been evaluated.

**Table 1:** Keywords for search databases.

	Mobile health			Total knee arthroplasty
	Mobile health			Total knee arthroplasty
OR	mHealth	**AND**	OR	TKA
OR	m-health		OR	Total knee replacement
OR	Smartphone		OR	TKR
OR	Mobile App*		OR	Knee replacements
			OR	Knee arthroplasty

### Inclusion criteria

We included articles in this review if they:

(1) Only considered full-text journal articles (we excluded abstracts);
(2) Were published in the English language;
(3) Were published during 2019–2023;
(4) Studies involving mobile health applications that provide telerehabilitation are implicated.

### Exclusion criteria

We excluded articles from this review if they:

(1) Were reviews review studies, editorials, conferences, brief reports, and dissertations;
(2) Not relevant to the research question;
(3) Studies that only reported patient and physician perspectives on smartphone apps show the importance of total knee replacement.
(4) Did not mention the design, development, implementation, and use of mobile health applications;
(5) Specifically reported guidelines, Strategies, policies, and challenges of mobile health applications for TKA and TKR patients.

### Screening and study selection

First, EndNote X21.2 (Thomson Reuters) software removed duplicate articles across databases. When a study was reported in multiple publications and presented the same data, we only retained the most recent version. Next, two researchers (F.S. and S.Z.) independently screened article titles and abstracts against inclusion and exclusion criteria. In the next step, the full text of selected articles was reviewed by researchers, and any disagreement was resolved by consensus between F.S. and S.Z. or by the decision of a third independent author. S2 Appendix provides information about excluded articles.

### Data extraction

Data from the selected studies were entered into a data extraction form designed according to the research objectives. Two authors reviewed the full-text articles separately and extracted all critical data from the studies. The extracted information included general information (author’s name, year of publication, and country), method (study design, study objective, type of Intervention, and TKA Measuring Tools), features of mHealth technology (type of Technology or Hardware, Output Data Format, and interoperability with other systems), and Type of Evaluation of mHealth app.

### Quality assessment

After screening the studies, the mixed methods appraisal tool (MMAT) was used to assess each study due to the variety of articles and existing methods. MMAT is an appropriate tool for evaluating all study designs, which decreases the risk of bias [9]. Therefore, the MMAT checklist was used to evaluate the articles. The quality of the studies was evaluated by F. S. and approved by S. Z., and disagreements were resolved through discussion. All the articles evaluated by the MMAT tool got the necessary score to participate in the study. The MMAT evaluation results are reported in the S3 Appendix; they are also mentioned in the last column of the S4 Appendix. S3 Appendix’s table includes the author’s name and year of publication, yes/no answers to 7 questions of the MMAT (Mixed Methods Appraisal Tool) questionnaire, and the score that each paper gets using the MMAT criteria.

## Result

**Fig 1:**
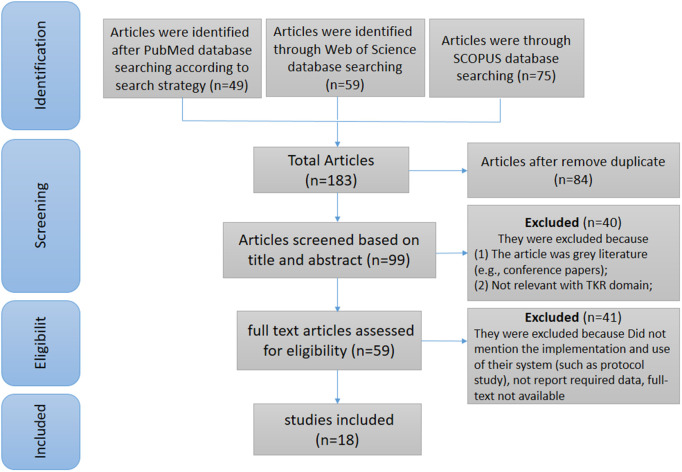
The PRISMA diagram.

After reviewing PubMed, Web of Science, SCOPUS, and Medline databases, 183 articles were retrieved. However, 84 items were removed as duplicates. Then, by screening the title and abstract of the studies, 59 articles were selected for full-text Screening, and 18 of them were included in this study. Fig 1 shows the PRISMA flow diagram (Preferred Reporting Item for Systematic Reviews and Meta-Analyses). This figure shows the steps of identifying, Screening, and qualifying the retrieved articles.

**Fig 2:**
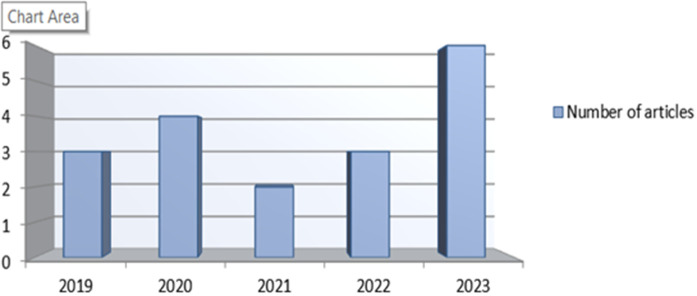
Distribution of articles by publication year.

We also categorized the selected articles according to the year of publication. Fig 2 shows the yearly distribution of published articles.

**Fig 3:**
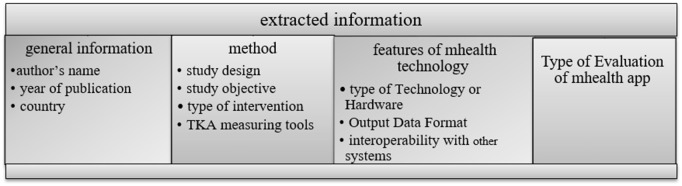
Subject of extracted information.

The information extracted from articles includes general information (year of publication, country), method (study design, objective, type of Intervention, and TKA Measuring Tools), features of mHealth technology (type of Technology or Hardware, Output Data Format), and Type of Evaluation of mHealth app. you can see the title of extracted information in Fig 3, you can also see the characteristics of selected articles in S4 Appendix. Also, you can see the summary of the characteristics of the selected articles in Table 2. The S4 Appendix’s columns include items extracted from the articles we mentioned earlier. These columns include the author’s name, country/year of publication, study design, study objective, type of Intervention, TKA measuring tools, type of technology or hardware, output data format, interoperability with other systems, type of evaluation, and MMAT score.

**Table 2:** The summary of selected articles’ characteristics.

	Percentage	Number	References
Study design			
RCT	50	9	[5,11–18]
Observational study	22.22	4	[19–22]
Pilot cohort	5.55	1	[23]
Quasi-experimental	5.55	1	[24]
Qualitative descriptive	5.55	1	[25]
Prospective parallel non-randomized trial	5.55	1	[26]
Mixed method	5.55	1	[27]
Country			
United State	27.77	5	[13,17,20,22,23]
Spain	16.66	3	[5,15,26]
Nederland	16.66	3	[14,18,24]
China	11.11	2	[11,25]
Ireland	11.11	2	[16,19]
Germany	5.55	1	[12]
Portugal	5.55	1	[27]
Taiwan	5.55	1	[21]
Type of Intervention			
Education	61.11	11	[5,13,14,17–19,20,23–25,27]
Treatment	61.11	11	[5,11,13,14,17–19,21,25–27]
Monitoring	50	9	[12,16,17,19,21–24,26]
Follow-up	33.33	6	[5,11,13–15,24]
Consultation	5.55	1	[26]
TKA measuring tools			
KOOS	33.33	6	[12,13,16–18,27]
PROMs	27.77	5	[11,17,22,23,25]
EQ-5D-5L	16.66	3	[12,13,18]
WOMAC	11.11	2	[15,16]
SF-12	5.55	1	[5]
Type of Technology or Hardware			
Sensor	44.44	8	[5,16,17,19,21,22,24,27]
Wearable activity tracker	22.22	4	[14,21–23]
Chatbot	16.66	3	[11,15,25]
Artificial intelligence	11.11	2	[15,27]
Camera Lenz	11.11	2	[18,19]
Smartwatch	11.11	2	[13,17]
Internet of things	5.55	1	[21]
Machine Learning	5.55	1	[23]
Type of Evaluation			
Effectiveness	61.11	11	[5,11,12,15,16,18,20,22–25]
Satisfaction	22.22	4	[13,18,26,27]
Cost-effectiveness	11.11	2	[5,14]
Engagement	11.11	2	[16,27]
Efficacy	5.55	1	[13]
Acceptability	5.55	1	[26]
Usability	5.55	1	[26]

RCT: Randomized controlled trials

KOOS: Knee Injury and Osteoarthritis Outcome Score

WOMAC: Western Ontario and McMaster Universities Osteoarthritis

PROMs: patient-reported outcome measures

EQ-5D-5L: European Quality of Life 5-Dimension 5-Level

SF-12: The Medical Outcomes Short-Form Health Survey

The United States, Spain, and the Netherlands were the most common countries that developed some telerehabilitation systems after TKA and studied their effectiveness. Each country accounted for 27.77% (5 studies), 16.66% (3 studies), and 16.66% (3 studies).

### 3.1. Study design

The study designs were randomized controlled trials (RCT), observational study, pilot cohort, quasi-experimental, qualitative descriptive, prospective parallel non-randomized trial, and mixed method type. Each of them accounted for 9 (50%), 4 (22.22%), 1 (5.55%), 1 (5.55%), 1 (5.55%), 1 (5.55%) and 1 (5.55%) studies, respectively.

### 3.2. Type of intervention

Most studies follow multiple goals of education, treatment, monitoring, follow-up, and consultation. Each of them accounted for 11 (61.11%), 11 (61.11%), 9 (50%), 6 (33.33%) and 1 (5.55%) of the studies, respectively.

### 3.3. TKA measuring tools

To evaluate patients after rehabilitation from total knee joint replacement, we use tools such as Knee Injury and Osteoarthritis Outcome Score (KOOS), patient-reported outcome measures (PROMs), European Quality of Life 5-Dimension 5-Level (EQ-5D-5L), Western Ontario and McMaster Universities Osteoarthritis (WOMAC) and The Medical Outcomes Short-Form Health Survey (SF-12). Each of them accounted for 6 (33.33%), 5 (27.77%), 3 (16.66%), 2 (11.11%) and 1 (5.55%) of the studies, respectively. In the following, we explain the most common tools used in articles.

### 3.4. Type of technology or hardware

In these studies, several pieces of equipment were usually used, such as sensors, wearable activity trackers, artificial intelligence, Chatbots, smartwatch, the Internet of Things, and Machine Learning. Each of them accounted for 8 (44.44%), 4 (22.22%), 3 (16.66%), 2 (11.11%), 2 (11.11%), 1 (5.55%) and 1 (5.55%) of the studies, respectively.

### 3.5. Type of evaluation of the mHealth app

Fifteen studies conducted an evaluation and published the results of this evaluation in the articles, and only three studies did not mention the evaluation. By conducting randomized control trial studies, telerehabilitation mobile applications or systems were evaluated [10]. Most of the evaluations have been done on effectiveness. The researchers have described and mentioned in their studies as effective, Satisfactory, cost-effective, engaging, efficient, acceptable, and Usable. Each of them accounted for 11 (61.11%), 4 (22.22%), 2 (11.11%), 2 (11.11%), 1 (5.55%), 1 (5.55%), and 1 (5.55%) of the studies, respectively.

## Discussion

Knee arthritis is common in the elderly, causes movement disorders, and affects daily tasks and quality of life. Some patients with knee arthritis need TKA surgery. Patients need to receive a suitable rehabilitation program to recover fully after surgery. The program lasts an average of 6–12 months [3]. Therefore, proper rehabilitation activities are necessary to return to daily life after TKA, but traditional physiotherapy imposes financial, time, and travel costs on patients, and this reduces Commitment to the physiotherapy program [4]. To reduce these costs and Increase Commitment, telerehabilitation is used as an alternative to traditional rehabilitation. Therefore, telerehabilitation technologies are considered innovative approaches. The present systematic review compared the studies that developed telerehabilitation systems and were published from 2019 to 2023.

As mentioned before, the MMAT tool was used to evaluate the studies. The MMAT tool helps reduce various biases in the research process by evaluating different aspects of a study, including research design, sampling, data collection, and data analysis. Questions related to research design help evaluate the selection of research methods and sampling, and reduce selection and information biases. Questions such as “Is randomization appropriately performed?” and “Are the measurements appropriate?” help evaluate the selection of samples and data collection, and reduce sampling and measurement biases. Finally, questions related to data analysis such as “Is the analysis appropriate to answer the research question?” help evaluate the analysis of data and reduce analysis biases. By using MMAT, researchers can ensure that their findings are based on strong and valid evidence, and thereby help reduce potential biases in research.

The most common tools used for system evaluation in articles are PROMs and KOOS. Each accounted for 7 (38.88%) and 3 (16.66%) of the studies. KOOS questionnaire is intended to be used in short-term intervals, for example, to evaluate changes from week to week due to treatment, and also for long-term intervals, for example, to evaluate a patient after years of arthritis; maybe this is the reason why it is widely used in studies [28]. Also, the PROM questionnaire is the most common tool used for system evaluation, enabling patients to report their quality of life, daily functioning, symptoms, and other aspects of their health and well-being. For this reason, it was widely used [29].

The KOOS scale was used in six studies to evaluate patients after TKA surgery. KOOS (Knee injury and Osteoarthritis Outcome Score) was developed as a tool to assess patients’ opinions about the knee and its related problems. The advantage of this questionnaire is the inclusion of two different subscales of physical performance related to daily life and sports and recreation. This demonstrates the tool’s validity for patients with a wide range of current and expected physical activity levels [28].

According to the S4 Appendix, some studies used other rehabilitation assessment methods, including measuring the amount of Patient-reported outcomes Measure (PROM). This evaluation method shows a person’s perception of their health through questionnaires. Answering the questions in this questionnaire will help hospitals and healthcare providers provide the care that patients need [29].

By reviewing the studies, it was found that five studies were conducted in the United States, and five studies were conducted in the Netherlands. The acceptance of telerehabilitation varies in other countries. Some countries, such as Canada, Australia, and the UK, have made progress, but developing countries face infrastructure and cultural challenges. In some countries, telerehabilitation is not considered a valid option for patient care and may be met with resistance from doctors and patients. In developing countries, technological infrastructure and access to the internet and digital devices are limited, which can hinder the adoption of telerehabilitation. Culture and language can also influence the adoption of telerehabilitation [30]. Therefore, for all the reasons mentioned above, the U.S. is more interested in using telerehabilitation than other countries. Regional differences in the adoption of telerehabilitation depend on various factors such as health policies, technological infrastructure, culture, and economy. In some regions, such as Asia and Africa, telerehabilitation may not be considered a valid option for patient care, while in other regions, such as Europe and North America, it is considered a valid option [31]. This review showed that developing countries have also used various technologies in recent years. This can be justified due to the benefits of telerehabilitation in reducing costs and its effectiveness. Telerehabilitation has become a valuable technology in this country, and it will be accepted by more and more people worldwide in the future [32].

Recovering from knee replacement surgery usually takes a long time. For this reason, the recovery period and care after surgery are of great importance, and in many cases, the patient is not followed up after surgery and does not have continuous access to a physician, and a solution must be found for necessary training and monitoring. The study results show that mobile health applications have many applications, including education, treatment, monitoring, follow-up, and consultation for patients after knee joint surgery. The most common use of telerehabilitation apps is in education. These apps are good tools for informing and educating patients about Essential information, such as disease conditions, dos and don’ts during rehabilitation, and how to perform therapeutic exercises. This information plays a significant role in facilitating rehabilitation so patients can achieve good health results.

One of the other important uses of these apps is in treatment. These apps can provide better healthcare to users and help with treatment by monitoring patients and facilitating communication with physicians. Also, the therapeutic goals of these apps are to improve motor performance, increase the range of motion, and reduce pain.

These apps can also be helpful by providing monitoring of patients’ condition by physicians. In patient monitoring, patient performance data is measured or collected over several weeks to several months, and the physician monitors each patient’s progress and adapts exercise plans and recommendations.

Some of these applications are used in the patient’s follow-up; the rehabilitation process is monitored in these applications, and the Correctness of performing exercises is checked. Also, consultations are provided to patients to explain their condition and ask their questions to the physician via teleconference. In the “objective section” of the S4 Appendix’s table, we specified the purpose of each study.

The most common hardware and technology used in the studies were sensors and chatbots. The sensor devices were used in eight studies. This tool can help telerehabilitation processes, for example, by measuring the range of motion of joints in three axes, checking the balance of the elderly during daily activities, and checking the amount and type of activity of patients during rehabilitation. For the reasons mentioned, the sensors are used to collect data from the simulation of the patient’s movements during exercises. After performing the movements, the physician or physiotherapist begins to analyze the data of the patient’s movements graphically and medically. Finally, the patient’s progress is evaluated at predetermined time intervals.

Three studies used chatbots to communicate with patients after TKA surgery. Chatbots are one of the important tools in the field of medicine, and they can communicate and interact with patients and provide healthcare services using advanced artificial intelligence algorithms. The applications of chatbots in rehabilitation include training and supporting patients, reminding and scheduling processes, monitoring and collecting medical data, and getting feedback.

The review of these studies showed that the programs that used new technologies, such as sensors, video conferencing, or wearable devices, reported engagement and satisfaction in their evaluations. These technologies have been increasingly used in this field due to their ability to provide personalized healthcare services, monitor patients, collect medical data, and educate and support patients. For this reason, these technologies have helped to improve treatment results. Also, studies that follow the three or more objectives of education, treatment, communication, motivation, follow-up, supervision, and counseling reported higher efficacy and the number of objectives and efficacy have a direct relationship.

Conducting evaluation is one of the most important parts of mobile health application development, because users are likely to gravitate toward products with better usability and recommend these products to others [33]. Usability means the user can do his work precisely and feel satisfied and comfortable while working with the product. For developers, usability is an important principle that determines the success and acceptance of an application [34].

Most studies performed randomized clinical trials on developed systems and evaluated the effectiveness of the application. Most evaluation methods in these studies determine the system’s acceptance, satisfaction, and usability. Several factors influence the usability and effectiveness of telerehabilitation systems after TKA surgery. These factors include simplicity of technology, education, communication, motivational mechanisms (appropriate feedback), usability issues, and patient engagement [35]. In the “evaluation of health app” section of the S4 Appendix’s table, we discussed the evaluation of the health app.

In addition, we examined the interoperability of apps with other health systems. The app was interoperable in ten studies with other EHR, EMR, and PHR systems. Electronic Health Records (EHR) or Electronic Medical Records (EMR) are systematic collections of electronic patient and community health information stored in digital format. These records can be shared across different healthcare facilities. PHR is an electronic program for recording personal medical information that is controlled by the patient and may be made available to healthcare providers [36]. With the help of EHR, you will have quick access to patient information such as medical records, diagnosis, type and name of disease, treatment, and test results. Because EHRs can store more data in a structured form, medical records can be more complete and improve patient care. However, the EHR should be designed so that it meets the needs of health care providers and they are not faced with a volume of additional information. Therefore, it is better to customize according to their needs [37]. Due to the advantages of EHR and EMR systems, the interoperability of apps with these systems increases their benefits, and when applications are integrated with EHR, their effectiveness increases significantly [38].

Some of these current mobile health applications for telerehabilitation have some acceptability and security problems, including Low acceptance of telerehabilitation technologies by patients and physicians, Limited effectiveness compared to traditional rehabilitation methods, Low patient participation in the telerehabilitation process, and Security concerns about the protection of medical data and doctor-patient communications. To address these limitations and increase the effectiveness of telerehabilitation systems, the following suggestions are made: 1-Using newer technologies: Using virtual reality instead of video conferencing to increase interaction and communication between the doctor and the patient. 2-Personalization: Adjusting the difficulty level of exercises based on the patient’s condition to increase the effectiveness of treatment. Personalization has been done in the study by Straat et al. [14] 3-Using additional equipment: Using suitable equipment to increase the effectiveness of treatments. A study by Sheridan et al. found that virtual assessment tools were effective in measuring knee flexion angle [19]. Also, a study by Tedesco et al. found that a wearable platform was effective in remotely monitoring knee rehabilitation [16]. A study by Tripuraneni et al. found that an innovative watch-based rehabilitation system effectively provided postoperative self-directed rehabilitation without compromising total knee arthroplasty outcomes [17]. 4- Gamification: Using gamification in presenting exercises to increase patient interest and interaction with the system. 5-Support from scientific and medical centers: Receiving support from scientific and medical centers to develop practical projects in telerehabilitation. 6-Solving security and communication problems: Trying to solve security, insurance, and communication problems, especially in developing countries, to increase the adoption and effectiveness of telerehabilitation. These barriers prevent the widespread adoption of technology or reduce the effectiveness of telerehabilitation, so they should be addressed quickly.

Also, the findings of telerehabilitation in TKA patients can be generalized to other types of rehabilitation or medical conditions. For instance, telerehabilitation can be applied to patients with stroke, spinal cord injuries, or chronic diseases such as diabetes or heart disease. Telerehabilitation in these conditions can provide similar benefits, including increased accessibility, reduced costs, and improved patient outcomes. Additionally, telerehabilitation can be tailored to meet the specific needs of different patient populations, making it a versatile and effective tool for rehabilitation. By expanding telerehabilitation to other rehabilitation areas, healthcare providers can improve the quality and accessibility of care, ultimately leading to better patient outcomes and improved healthcare systems.

Based on the findings of this review, several research directions are suggested to address the gaps in telerehabilitation: 1-Investigate telerehabilitation’s effectiveness in various medical conditions. 2-Develop customized telerehabilitation models. 3-Examine telerehabilitation’s impact on patients’ quality of life. 4-Conduct economic evaluations of telerehabilitation. 5-Develop new technologies for telerehabilitation. 5-Investigate security and privacy concerns in telerehabilitation. 6-Establish telerehabilitation more standards. These research directions can help improve our understanding of telerehabilitation and develop more effective programs to improve patient outcomes.

## Conclusion

Telerehabilitation can be as effective as traditional rehabilitation in improving the condition of patients after TKA. However, it is suggested that improvements should always be made to achieve better results of telerehabilitation. This will become possible due to the rapid development of technologies.

However, patients and therapists are satisfied with telerehabilitation. It uses information and communication technology to provide high-quality, low-cost, continuous treatments. Shortly, it will be considered as a new method to meet all rehabilitation needs of these patients.

## Supporting information

S1 Appendix Search strategy. (PDF)

S2 Appendix Studies excluded from the review. (PDF)

S3 Appendix MMAT evaluation results. (PDF)

S4 Appendix Characteristics of selected articles. (PDF)
