# Supplementary material for: Utilization of telerehabilitation in TKR patients: A systematic review
Source: PLoS One. 2025 Jul 23;20(7):e0324074. doi: 10.1371/journal.pone.0324074 (PMC12286395; doi:10.1371/journal.pone.0324074)
Supplement: S1 Appendix — (PDF) [file pone.0324074.s001.pdf]

S1 Appendix: Search strategy

Database	Search String
PubMed	(mobile health[Title/Abstract] OR Mhealth[Title/Abstract] OR m-health[Title/Abstract] OR Smartphone[Title/Abstract] OR "Mobile App"[Title/Abstract]) AND (total knee arthroplasty[MeSH] OR TKA[Title/Abstract] OR "total knee replacement"[Title/Abstract] OR TKR[Title/Abstract] OR "knee replacements"[Title/Abstract] OR "knee arthroplasty"[MeSH])
Scopus	(TITLE-ABS-KEY((mobile health OR Mhealth OR m-health OR Smartphone OR "Mobile App*")) AND TITLE-ABS-KEY((total knee arthroplasty OR TKA OR "total knee replacement" OR TKR OR "knee replacements" OR "knee arthroplasty"))
Web of Science	TS=(mobile health OR Mhealth OR m-health OR Smartphone OR "Mobile App*") AND TS=(total knee arthroplasty OR TKA OR "total knee replacement" OR TKR OR "knee replacements" OR "knee arthroplasty")
