# Supplementary material for: Utilization of telerehabilitation in TKR patients: A systematic review
Source: PLoS One. 2025 Jul 23;20(7):e0324074. doi: 10.1371/journal.pone.0324074 (PMC12286395; doi:10.1371/journal.pone.0324074)
Supplement: S2 Appendix — (PDF) [file pone.0324074.s002.pdf]

S2 Appendix: studies excluded for review

Number	Title	Authors (et al.)	Published Year	Reason for Exclusion
1	Advanced rehabilitation technology in orthopaedics-a narrative review	Y. Kuroda	2021	Review
2	The effectiveness of internet-based telerehabilitation among patients after total joint arthroplasty: An integrative review	Q. Wang	2021	Review
3	eHealth in Geriatric Rehabilitation: Systematic Review of Effectiveness, Feasibility, and Usability	J. J. M. Kraaijkamp	2021	Systematic Review
4	Expanding Role of Technology in Rehabilitation After Lower-Extremity Joint Replacement: A Systematic Review	J. F. McKeon	2021	Systematic Review
5	Frequency and Outcomes of Preoperative Stress Testing in Total Hip and Knee Arthroplasty from 2004 to 2017	D. S. Rubin	2021	Systematic Review
6	Integrated care programmes for sport and work participation, performance of physical activities and quality of life among orthopaedic surgery patients: a systematic review with meta-analysis	P. Coenen	2020	Systematic Review and Meta-analysis
7	Obesity, preoperative weight loss, and telemedicine before total joint arthroplasty: a review	M. W. Seward	2022	Review
8	Smartphone apps for total hip replacement and total knee replacement surgery patients: a systematic review	H. C. Van Dijk-Huisman	2020	Systematic Review
9	The use of mHealth in orthopedic surgery: A scoping review	S. Dionisi	2021	scoping review
10	Cognitive Training for Robotic Arm-Assisted Unicompartmental Knee Arthroplasty through a Surgical Simulation Mobile Application	G. L. Vestermark	2019	Having different scope compared to the research question
11	Concept verification of a Remote Automatic Scoring System for Evaluating Knee Function after Total Knee Arthroplasty	H. Zhang	2021	Having a limited scope compared to the research question
12	Connected orthopedics and trauma surgery: New perspectives	N. Reina	2019	Not relevant with TKR domain
13	CORR Insights®: Preoperative Physical Therapy Education Reduces Time to Meet Functional Milestones after Total Joint Arthroplasty	C. J. Marques	2018	Having a limited scope compared to the research question

14	Cost-effective mobile-based healthcare system for managing total joint arthroplasty follow-up	M. Bitsaki	2017	Didn’t mentioned to mobile app implementation
15	Current Practice Trends in Primary Hip and Knee Arthroplasty Among Members of the American Association of Hip and Knee Surgeons: An Update During the COVID-19 Pandemic	M. P. Abdel	2021	Having different scope compared to the research question (focus on Practice aspect)
16	Early outcomes of primary total hip arthroplasty with use of a smartphone-based care platform: a prospective randomized controlled trial	D. A. Crawford	2021	not reporting required data about mobile app
17	Enhanced recovery after surgery (Eras) for hip and knee replacement—why and how it should be implemented following the covid-19 pandemic	T. W. Wainwright	2021	not reporting required data about mobile app
18	Evaluation of benefits and accuracy of a mobile application in planning total knee arthroplasty	J. B. S. Nogueira	2018	not reporting required data about mobile app
19	The Impact of Painless Navigation in Conventionally Aligned Total Knee Arthroplasty	P. Koenen	2018	Having a limited scope compared to the research question
20	Master techniques in orthopedic surgery: Knee arthroplasty	M. W. Pagnano	2018	Having different scope compared to the research question (focus on surgical aspect)
21	Monitoring the kinematics of Walking and Running Gait after total knee replacement using a generation of Kinematic Retaining prosthetic knee implant	S. Noroozi	2021	Having a limited scope compared to the research question
22	Movement pattern biofeedback training after total knee arthroplasty: Randomized clinical trial protocol	M. J. Bade	2020	Having a limited scope compared to the research question
23	Orthopaedic: Knowledge update: Hip and knee reconstruction	M. A. Mont	2018	Having different scope compared to the research question (focus on surgical aspect)
24	Patient-reported outcomes feedback report for knee arthroplasty patients should present selective information in a simple design - findings of a qualitative study	Y. Wang	2021	Having a limited scope compared to the research question
25	Planning a total knee arthroplasty through an application for mobile devices: case report	J. B. S. Nogueira	2018	not reporting required data about mobile app

26	Preferences and motivation for weight loss among knee replacement patients: Implications for a patient-centered weight loss intervention	C. A. Pellegrini	2017	Having a limited scope compared to the research question
27	Quantifying the Surgeon's Increased Burden of Postoperative Work for Modern Arthroplasty Surgery	R. P. Shah	2021	Having different scope compared to the research question (focus on surgical aspect)
28	Role of mobile applications for the success of enhanced recovery after surgery programme	A. Nair	2021	mentioned to role - not mentioned to mobile app implementation
29	Secure Processing of Stream Cipher Encrypted Data Issued from IOT: Application to a Connected Knee Prosthesis	M. Pistono	2019	mentioned to infrastructure - not mentioned to mobile app implementation
30	Smartphone assessment of knee flexion compared to radiographic standards	M. J. Dietz	2017	Having a limited scope compared to the research question
31	Smartphone technology: a reliable and valid measure of knee movement in knee replacement	H. Castle	2018	Having a limited scope compared to the research question
32	A social media–promoted educational community of joint replacement patients using the WeChat app: Survey study	X. Z. Zhang	2021	Having a limited scope compared to the research question
33	A Step in the Right Direction: Body Location Determines Activity Tracking Device Accuracy in Total Knee and Hip Arthroplasty Patients	R. Goel	2020	Having a limited scope compared to the research question
34	Thermographic assessment of reperfusion profile following using a tourniquet in total knee arthroplasty: A prospective observational	M. Alisi	2021	Having a limited scope compared to the research question
35	Using mHealth App to Support Treatment Decision-Making for Knee Arthritis: Patient Perspective	H. Zheng	2017	Having different scope compared to the research question (Support Treatment Decision-Making)
36	When can I go home after my knee replacement? Factors affecting the duration of in-hospital stay after knee replacement	V. Khanna	2019	Having different scope compared to the research

				question (duration of in-hospital stay)
37	2021 Mark Coventry Award: Use of a smartphone-based care platform after primary partial and total knee arthroplasty: a prospective randomized controlled trial	D. A. Crawford	2021	not reporting required data about mobile app
38	Augmented Reality-Assisted Femoral Bone Resection in Total Knee Arthroplasty	S. Tsukada	2021	Having different scope compared to the research question (Augmented Reality-Assisted)
39	Augmented reality-based navigation system applied to tibial bone resection in total knee arthroplasty	S. Tsukada	2019	Having different scope compared to the research question (Augmented Reality-Assisted)
40	App-based rehabilitation program after total knee arthroplasty: a randomized controlled trial	H. C. Bäcker	2021	not reporting required data about mobile app
41	Assessing the knee flexion range of motion after total knee arthroplasty: Technology versus senses	U. Z. Kocak	2021	Having a limited scope compared to the research question (only knee flexion range)
42	Clinician perceptions of a prototype wearable exercise biofeedback system for orthopaedic rehabilitation: A qualitative exploration	R. Argent	2018	not reporting required data about mobile app
43	The digital patient journey solution for patients undergoing elective hip and knee arthroplasty: Protocol for a pragmatic randomized controlled trial	M. Jansson	2020	not reporting required data about mobile app
44	Effect of a smartphone app plus an accelerometer on physical activity and functional recovery during hospitalization after orthopedic surgery	K. F. Mateo	2020	not reporting required data about mobile app
45	Effectiveness of a Mobile eHealth App in Guiding Patients in Pain Control and Opiate Use After Total Knee Replacement: Randomized Controlled Trial	Y. Pronk	2020	Having a limited scope compared to the research question (only Pain Control)
46	The effectiveness of the use of a digital activity coaching system in addition to a two-week home-based exercise program in patients after total knee arthroplasty: study protocol for a randomized controlled trial	K. E. M. Harmelink	2017	Having a limited scope compared to the research question (two-week home-based exercise program)
47	Improving patient outcomes following total joint arthroplasty: Is there an app for that?	J. I. Wolfstadt	2019	not mentioned to mobile app implementation

48	An Intelligent Remote Monitoring System for Total Knee Arthroplasty Patients	H. Scheper	2019	Having a limited scope compared to the research question (only Remote Monitoring)
49	Knee Flexion Angle Measurement Using Virtual Assessment Tools: Correct Procedure and Potential Pitfalls	G. A. Sheridan	2020	Having a limited scope compared to the research question (only Knee Flexion Angle Measurement)
50	A mobile app for postoperative wound care after arthroplasty: Ease of use and perceived usefulness	Y. Msayib	2017	Having a limited scope compared to the research question (only wound care)
51	Monitoring and assessment of rehabilitation progress on range of motion after total knee replacement by sensor-based system	Y. P. Huang,	2020	Having a limited scope compared to the research question (only range of motion)
52	Monitoring Patient Recovery After THA or TKA Using Mobile Technology	S. Lyman	2020	Having a limited scope compared to the research question (only Monitoring)
53	Monitoring Surgical Incision Sites in Orthopedic Patients Using an Online Physician-Patient Messaging Platform	J. Zhang	2019	Having a limited scope compared to the research question (only Monitoring Surgical Incision Sites in Orthopedic Patients)
54	A monitoring system for walking rehabilitation after THR or TKR surgeries	Z. Qianpeng	2017	Having a limited scope compared to the research question (only walking rehabilitation)
55	A novel, automated text-messaging system is effective in patients undergoing total joint arthroplasty	K. J. Campbell	2018	Having a limited scope compared to the research question (automated text-messaging system)
56	Prospective Validation of a Demographically Based Primary Total Knee Arthroplasty Size Calculator	R. A. Sershon	2019	Having different scope compared to the research

				question (focus on surgical aspect)
57	Readability of Information on Smartphone Apps for Total Hip Replacement and Total Knee Replacement Surgery Patients	S. Bahadori	2020	mentioned to benefit - not mentioned to mobile app implementation
58	Reducing sedentary time using an innovative mHealth intervention among patients with total knee replacement: Rationale and study protocol	C. A. Pellegrini	2021	study protocol - not mentioned to mobile app implementation
59	Reliability of the Knee Smartphone-Application Goniometer in the Acute Orthopedic Setting	L. C. Pereira	2017	Having different scope compared to the research question (use app to help with surgical aspect)
60	Reliability, Concurrent Validity, and Minimal Detectable Change for iPhone Goniometer App in Assessing Knee Range of Motion	S. P. Mehta	2017	Having different scope compared to the research question (use app to help with surgical aspect)
61	Remote Patient Monitoring Using Mobile Health for Total Knee Arthroplasty: Validation of a Wearable and Machine Learning–Based Surveillance Platform	P. N. Ramkumar	2019	Having different scope compared to the research question (Machine Learning–Based Surveillance Platform)
62	The safety, efficacy and cost-effectiveness of the Maxm Skate, a lower limb rehabilitation device for use following total knee arthroplasty: Study protocol for a randomised controlled trial	M. G. Liptak	2019	study protocol - mentioned to safety, efficacy - not mentioned to mobile app implementation
63	Smartphone App with an Accelerometer Enhances Patients' Physical Activity Following Elective Orthopedic Surgery: A Pilot Study	H. C. van Dijk-Huisman	2020	Having a limited scope compared to the research question (Enhances Patients' Physical Activity)
64	Smartphone goniometer has excellent reliability between novice and experienced physical therapists in assessing knee range of motion	S. P. Mehta	2021	Having a limited scope compared to the research question (only assessing knee range of motion)

65	A Smartwatch Paired With A Mobile Application Provides Postoperative Self-Directed Rehabilitation Without Compromising Total Knee Arthroplasty Outcomes: A Randomized Controlled Trial	K. R. Tripuraneni	2021	not reporting required data about mobile app
66	Telerehabilitation program for older adults on a waiting list for physical therapy after hospital discharge: study protocol for a pragmatic randomized trial protocol	P. R. T. Borges	2021	study protocol - not mentioned to mobile app implementation
67	Use of an App-Controlled Neuromuscular Electrical Stimulation System for Improved Self-Management of Knee Conditions and Reduced Costs	M. Chughtai	2017	Having a limited scope compared to the research question (only Neuromuscular Electrical Stimulation System)
68	Using smartphone-based accelerometers to gauge postoperative outcomes in patients with NPH: Implications for ambulatory monitoring	C. Sprau	2021	not reporting required data about mobile app
69	The Virtual Foot and Ankle Physical Examination	S. K. Eble	2020	Having a limited scope compared to the research question (only Foot and Ankle Physical Examination)
70	Wearable sensor-based exercise biofeedback for orthopaedic rehabilitation: A mixed methods user evaluation of a prototype system	R. Argent	2019	not reporting required data about mobile app
71	Wearable sensors for remote patient monitoring in orthopedics	R. D. Gurchiek	2021	Having a limited scope compared to the research question (only have Wearable sensors)
72	Artificial Intelligence and Arthroplasty at a Single Institution: Real-World Applications of Machine Learning to Big Data, Value-Based Care, Mobile Health, and Remote Patient Monitoring	P. N. Ramkumar	2019	Having different scope compared to the research question (Artificial Intelligence)
73	SEKO: Smart system for assisting home-based rehabilitation of knee arthroplasty patients	A. Penders	2018	not reporting required data about mobile app
74	Unicompartmental knee arthroplasty results in a better gait pattern than total knee arthroplasty: Gait analysis with a smartphone application	D. Çankaya	2021	Having a limited scope compared to the research question (only Gait analysis)
75	Virtual reality rehabilitation following total knee arthroplasty: a systematic review and meta-analysis of randomized controlled trials	Gazendam	2022	Systematic Review and Meta-analysis

76	Digital Rehabilitation after Knee Arthroplasty: A Multi-Center Prospective Longitudinal Cohort Study	J. Lebleu	2023	not reporting required data about mobile app
77	A Web-Based Communication Tool for Postoperative Follow-up and Pain Assessment at Home After Primary Knee Arthroplasty: Feasibility and Usability Study	T. Rian	2022	Having a limited scope compared to the research question
78	Mobile Applicant's Effect on Patient Satisfaction and Compliance in Total Joint Arthroplasty: A Systematic Review and Meta-analysis	R. Monárrez	2023	Systematic Review and Meta-analysis
79	Using Telehealth to Guarantee the Continuity of Rehabilitation during the COVID-19 Pandemic: A Systematic Review	E. Brigo	2022	Systematic Review
80	Video Analysis of Communication by Physiotherapists and Patients in Video Consultations: A Qualitative Study using Conversation Analysis	L. M. Seuren	2023	Having a limited scope compared to the research question
81	Face-to-face and telerehabilitation delivery of circuit training have similar benefits and acceptability in patients with knee osteoarthritis: a randomised trial	J. B. Aily	2023	Not relevant with TKR domain

After reviewing databases, 183 articles were retrieved, then duplicate items (84 items) were removed and the title and abstract of studies were screened, 40 items were Excluded because they was grey literature or not relevant to TKR surgery and 59 articles were remained for full text Screening and after full-text screening, 41 articles were Excluded and 18 articles were included in this study and analyzed.
