# Supplementary material for: Utilization of telerehabilitation in TKR patients: A systematic review
Source: PLoS One. 2025 Jul 23;20(7):e0324074. doi: 10.1371/journal.pone.0324074 (PMC12286395; doi:10.1371/journal.pone.0324074)
Supplement: S3 Appendix — (PDF) [file pone.0324074.s003.pdf]

S3 Appendix: Quality assessment using MMAT (Mixed Methods Appraisal Tool), version 2018

**Qualitative**								
Authors/years	S1. Are there clear research questions?	S2. Do the collected data allow to address the research questions?	1.1. Is the qualitative approach appropriate to answer the research question?	1.2. Are the qualitative data collection methods adequate to address the research question?	1.3. Are the findings adequately derived from the data?	1.4. Is the interpretation of results sufficiently substantiated by data?	1.5. Is there coherence between qualitative data sources, collection, analysis and interpretation?	Score (%)
Qingling Wang et al. (July 2023)	YES	YES	YES	YES	YES	YES	YES	100

**Quantitative randomized controlled trials**								
Authors/years	S1. Are there clear research questions?	S2. Do the collected data allow to address the research questions?	2.1. Is randomization appropriately performed?	2.2. Are the groups comparable at baseline?	2.3. Are there complete outcome data?	2.4. Are outcome assessors blinded to the intervention provided?	2.5 Did the participants adhere to the assigned intervention?	Score (%)
Qingling Wang et al. (April 2023)	YES	YES	YES	YES	YES	NO	YES	80
Viktoria Steinbeck et al. (2023)	YES	YES	YES	YES	YES	NO	YES	80
Jacob Alexander et al. (2023)	YES	YES	YES	YES	YES	Can’t tell	YES	80
A. Carlien Straat et al. (2023)	YES	YES	YES	YES	YES	YES	YES	100
José-María Blasco et al. (2023)	YES	YES	YES	YES	YES	YES	YES	100
Jordi Colombia et al. (202١)	YES	YES	YES	YES	YES	Can’t tell	YES	80
Krishna R Tripuraneni et al. (2021)	YES	YES	YES	YES	YES	Can’t tell	YES	80
Thomas Timmers et al. (2019)	YES	YES	YES	YES	YES	YES	YES	100

Fernando Dias Correia et al. (2019)	YES	YES	YES	YES	YES	YES	YES	100
**Quantitative non-randomized**								
Authors/years	S1. Are there clear research questions?	S2. Do the collected data allow to address the research questions?	3.1. Are the participants representative of the target population?	3.2. Are measurements appropriate regarding both the outcome and intervention (or exposure)?	3.3. Are there complete outcome data?	3.4. Are the confounders accounted for in the design and analysis?	3.5. During the study period, is the intervention administered (or exposure occurred) as intended?	Score (%)
Gerard A. Sheridan et al. (202٢)	YES	YES	YES	YES	YES	YES	YES	100
Salvatore Tedesco et al. (202٢)	YES	YES	YES	YES	YES	YES	YES	100
Gerard Torres et al. (2020)	YES	YES	YES	YES	YES	YES	YES	100
van Dijk-Huisman et al. (2020)	YES	YES	YES	YES	YES	YES	YES	100
Yo-Ping Huang et al. (202٠)	YES	YES	YES	YES	YES	YES	YES	100
Stephen Lyman et al. (202٠)	YES	YES	YES	YES	YES	YES	YES	100
Prem Ramkumar et al. (2019)	YES	YES	YES	YES	YES	YES	YES	100
**Quantitative descriptive**								
Authors/years	S1. Are there clear research questions?	S2. Do the collected data allow to address the research questions?	4.1. Is the sampling strategy relevant to address the research question?	4.2. Is the sample representative of the target population?	4.3. Are the measurements appropriate?	4.4. Is the risk of nonresponse bias low?	4.5. Is the statistical analysis appropriate to answer the research question?	Score (%)
Carly E Milliren et al. (202٢)	YES	YES	YES	YES	YES	Can’t tell	YES	80
