# Supplementary material for: Utilization of telerehabilitation in TKR patients: A systematic review
Source: PLoS One. 2025 Jul 23;20(7):e0324074. doi: 10.1371/journal.pone.0324074 (PMC12286395; doi:10.1371/journal.pone.0324074)
Supplement: S4 Appendix — (PDF) [file pone.0324074.s004.pdf]

S4 Appendix's Table: A Summary Form of Selected articles

Author's name	Country / year	Study Design	Study Objective	Type of Intervention	TKA Measuring Tools	Type of Technology or Hardware	Output Data Format	Interoperability with other Systems	Type of Evaluation	MMAT Score
Qingling Wang [10]	China (2023)	Randomized controlled trial	This study aimed to evaluate the effectiveness of a rehabilitation program delivered via a mobile application among Chinese patients after total hip or knee arthroplasty.	Treatment, Follow-up	PROMs (Patient-reported outcomes Measure)	Chatbot	Text, photo, video	YES	Effectiveness	5/5
Viktoria Steinbeck [11]	Germany (2023)	Randomized controlled trial	To determine whether PROM monitoring can improve health outcomes for patients with joint replacement compared with the standard of care	Monitoring	EQ-5D-5L (European Quality of Life 5-Dimension 5-Level), KOOS PS	Email	Text, photo	YES	Effectiveness	4/5
Jacob S. Alexander [12]	US (2023)	Prospective Randomized controlled trial	Determine the non-inferiority of one such system after primary knee arthroplasty as compared to traditional in-person physiotherapy	Education, Treatment, Follow-up	KOOS JR, EQ-5D-5L	Smart watch	Text, photo, video	YES	Efficacy, Satisfaction	4/5

S4 Appendix's Table: A Summary Form of Selected articles

Author's name	Country / year	Study Design	Study Objective	Type of Intervention	TKA Measuring Tools	Type of Technology or Hardware	Output Data Format	Interoperability with other Systems	Type of Evaluation	MMAT Score
A. Carlien Straat [13]	Netherland (2023)	Multicenter Randomized controlled trial	Describe the development, content and protocol of study that assesses a perioperative integrated care program, including a personalized e-Health app, for knee TKA patients.	Education, Treatment, Follow-up	PROMIS-PF (Patient-reported outcomes Measurement Information System)	Activity Tracker	Text, chart and graph, video	N/A	Cost-Effectiveness	5/5
Qingling Wang [14]	China (2023)	Qualitative descriptive study	This study aimed to investigate patients' experiences using a mobile app-based rehabilitation program that was developed for patients after THA and TKA	Education, Treatment	Patient-reported physical function	Chatbot	Text, photo, video	YES	Effectiveness	4/5
José-Maria Blasco [15]	Spain (2023)	Randomized controlled trial	Aim to determining the effectiveness of using a virtual assistant (i.e., Chabot)	Follow-up	WOMAC	Chatbot, Artificial Intelligence	Text, photo, video	N/A	Effectiveness	5/5
Gerard A. Sheridan [16]	Ireland (2022)	Observational study	Impact of observer position relative to the flexed knee on the perceived angle measure	Education, Treatment, Monitoring	Sensor (to measure the extent of knee flexion), Camera lens (to check knee movements)	Sensor, Camera Lenz	Text, video, Sensor output	YES	N/A	5/5

S4 Appendix's Table: A Summary Form of Selected articles

Author's name	Country / year	Study Design	Study Objective	Type of Intervention	TKA Measuring Tools	Type of Technology or Hardware	Output Data Format	Interoperability with other Systems	Type of Evaluation	MMAT Score
Salvatore Tedesco [17]	Ireland (2022)	Prospective, pragmatic trial	A platform for the remote assessment of patients undergoing knee rehabilitation with the aim of capturing real-time objective assessment of physical rehabilitation exercises and support clinicians in decision-making	Monitoring	KOOS, IKDC, WOMAC	Sensor	Text, photo, chart and graph, video, Sensor output	YES	Effectiveness, Engagement	5/5
Carly E Milliren [18]	US (2022)	Observational study	Improve surgical cancellation and post-surgical outcomes for adult patients undergoing TKA	Education	Length of stay, frequent hospital revisits and readmission	Email	Text	YES	Effectiveness	4/5
Jordi Colombia [5]	Spain (2021)	Prospective, 2-arm, parallel trial	Aim to assess the effectiveness and cost-effectiveness of implementing a mobile health (mHealth)-enabled IC model for patients undergoing primary THA or TKA.	Education, Treatment, Follow-up	SF-12 (12-item short-form survey)	Sensor, mapping function	Text, chart and graph, location on the map, Sensor output	YES	Effectiveness, Cost-Effectiveness	4/5

S4 Appendix's Table: A Summary Form of Selected articles										
Author's name	Country / year	Study Design	Study Objective	Type of Intervention	TKA Measuring Tools	Type of Technology or Hardware	Output Data Format	Interoperability with other Systems	Type of Evaluation	MMAT Score
Krishna R Tripuraneni [19]	US (2021)	Randomized controlled trial	The purpose of this study was to determine if there was an impact on postoperative outcomes with the use of an SDR (Self-directed rehabilitation) program after primary TKA.	Education, Treatment, Monitoring	KOOS, PROMs, Measurement of knee range of motion	Sensor, smart watch	Text, video, Sensor output	N/A	N/A	4/5
Gerard Torres [20]	Spain (2020)	Prospective, pragmatic, 2-arm, parallel trial	Aim to assess the acceptability, usability, and satisfaction of a mhealth-enabled IC model for complex chronic patients in both patients and health professionals	Treatment, Monitoring and Consultation	P3CEQ, NCQ (both measuring person-centeredness and continuity of care)	Mapping function	Text, chart and graph, location on the map	YES	Usability, Acceptability, Satisfaction	5/5
Yo-Ping Huang [21]	Taiwan (2020)	Observational study	This study proposes a sensor-based system for effectively monitoring rehabilitation	Treatment, Monitoring	Sensor (to measure the extent of knee flexion after TKA surgery)	Sensor, wearable devices, Internet of Things	Text, video, Sensor output	N/A	N/A	5/5

S4 Appendix's Table: A Summary Form of Selected articles

Author's name	Country / year	Study Design	Study Objective	Type of Intervention	TKA Measuring Tools	Type of Technology or Hardware	Output Data Format	Interoperability with other Systems	Type of Evaluation	MMAT Score
van Dijk-Huisman HC[22]	Netherlands (2020)	Non-randomized Quasi-experimental	Developed Hospital Fit; which is a smartphone application with an accelerometer, designed for hospitalized patients. It enables objective activity monitoring and provides patients with insights into their recovery progress and offers a tailored exercise program. This study was investigate the potential of Hospital Fit to enhance PA levels and functional recovery following orthopedic surgery	Education, Monitoring, Follow-up	POD1 (Modified Iowa Level of Assistance Scale for Assessment functional recovery)	Accelerometer Sensor	Text, photo video	N/A	Effectiveness	5/5
Stephen Lyman [23]	US (2020)	Observational study	We investigate the feasibility of using mobile technology to collect daily step data and biweekly PROMs	Monitoring	PROMs, Counting step numbers	Accelerometer Sensor, Wearable devices	Text, video, Sensor output	YES	Effectiveness	5/5

S4 Appendix's Table: A Summary Form of Selected articles										
Author's name	Country / year	Study Design	Study Objective	Type of Intervention	TKA Measuring Tools	Type of Technology or Hardware	Output Data Format	Interoperability with other Systems	Type of Evaluation	MMAT Score
Prem N Ramkumar[24]	U.S (2019)	Cohort	Validate the feasibility of a remote patient monitoring (RPM) system	Education, Monitoring	PROMs, Measurement of knee range of motion	Machine Learning, Wearable knee sleeve	Text, photo, video	N/A	Effectiveness	5/5
Thomas Timmers [25]	Netherlands (2019)	Randomized controlled trial	Determine whether actively educating TKR patients with timely, day-to-day postoperative care information through an app could lead to a decrease in their level of pain compared to those who only receive standard information about their recovery through the app.	Education, Treatment	KOOS PS, EQ-5D, ability to perform daily work	Camera Lenz	Text, photo, video	N/A	Effectiveness, Satisfaction	5/5

S4 Appendix’s Table: A Summary Form of Selected articles

Author’s name	Country / year	Study Design	Study Objective	Type of Intervention	TKA Measuring Tools	Type of Technology or Hardware	Output Data Format	Interoperability with other Systems	Type of Evaluation	MMAT Score
Fernando Dias Correia [26]	Portugal (2019)	Prospective, parallel-group feasibility study	Follow-up results of a feasibility study comparing two different home-based programs after TKA: conventional face-to-face sessions and a digital intervention performed through the use of an artificial intelligence-powered biofeedback system under remote clinical monitoring.	Education, Treatment	KOOS, Measurement of knee range of motion	Sensor, Web portal, Artificial Intelligence	Text, photo, video, Sensor output	N/A	Engagement, Satisfaction	5/5
